# Human exposure to synthetic endocrine disrupting chemicals (S-EDCs) is generally negligible as compared to natural compounds with higher or comparable endocrine activity: how to evaluate the risk of the S-EDCs?

**DOI:** 10.1007/s00204-020-02800-8

**Published:** 2020-06-08

**Authors:** Herman Autrup, Frank A. Barile, Sir Colin Berry, Bas J. Blaauboer, Alan Boobis, Herrmann Bolt, Christopher J. Borgert, Wolfgang Dekant, Daniel Dietrich, Jose L. Domingo, Gio Batta Gori, Helmut Greim, Jan Hengstler, Sam Kacew, Hans Marquardt, Olavi Pelkonen, Kai Savolainen, Pat Heslop-Harrison, Nico P. Vermeulen

**Affiliations:** 1 Institute of Public Health, University of Aarhus, Aarhus, Denmark; 2 College of Pharmacy and Health Sciences, St John’s University, Queens, NY USA; 3 Queen Mary University of London, London,, UK; 4 Division of Toxicology, Institute for Risk Assessment Sciences, Utrecht University, Utrecht, The Netherlands; 5 National Heart and Lung Institute, Imperial College, London, UK; 6 Leibniz Research Centre for Working Environment and Human Factors (IfADo), TU Dortmund, Dortmund, Germany; 7 Applied Pharmacology and Toxicology, Inc., Gainesville, FL USA; 8 Department of Toxicology, University of Wuerzburg, Wuerzburg, Germany; 9 Human and Environmental Toxicology, University of Konstanz, Konstanz, Germany; 10 Laboratory of Toxicology and Environmental Health, School of Medicine, IISPV, Universitat ‘Rovira I Virgili’, Reus, Spain; 11 The Health Policy Center, Bethesda, MD USA; 12 McLaughlin Centre for Risk Assessment, University of Ottawa, Ottawa, Canada; 13 Toxicology, Hamburg, Germany; 14 Department of Pharmacology and Toxicology, University of Oulu, Oulu, Finland; 15 Nanosafety Research Centre, Finnish Institute of Occupational Health, Helsinki, Finland; 16 Department of Genetics and Genome Biology, University of Leicester, Leicester, UK; 17 Department of Chemistry and Pharmaceutical Sciences, Vrije Universiteit, Amsterdam, The Netherlands; 18 Technical University of Munich, Hohenbachernstrasse 15-17, 85350 Freising-Weihenstephan, Germany

**Keywords:** Endocrine disruption, Risk characterisation, Testing

## Abstract

Theoretically, both synthetic endocrine disrupting chemicals (S-EDCs) and natural (exogenous and endogenous) endocrine disrupting chemicals (N-EDCs) can interact with endocrine receptors and disturb hormonal balance. However, compared to endogenous hormones, S-EDCs are only weak partial agonists with receptor affinities several orders of magnitude lower. Thus, to elicit observable effects, S-EDCs require considerably higher concentrations to attain sufficient receptor occupancy or to displace natural hormones and other endogenous ligands. Significant exposures to exogenous N-EDCs may result from ingestion of foods such as soy-based diets, green tea and sweet mustard. While their potencies are lower as compared to natural endogenous hormones, they usually are considerably more potent than S-EDCs. Effects of exogenous N-EDCs on the endocrine system were observed at high dietary intakes. A causal relation between their mechanism of action and these effects is established and biologically plausible. In contrast, the assumption that the much lower human exposures to S-EDCs may induce observable endocrine effects is not plausible. Hence, it is not surprising that epidemiological studies searching for an association between S-EDC exposure and health effects have failed. Regarding testing for potential endocrine effects, a scientifically justified screen should use in vitro tests to compare potencies of S-EDCs with those of reference N-EDCs. When the potency of the S-EDC is similar or smaller than that of the N-EDC, further testing in laboratory animals and regulatory consequences are not warranted.

## Introduction

November 7, 2018 the European Commission has published the following document: Communication from the Commission to the European Parliament, the Council, the European Economic and Social Committee and the Committee of the Regions: towards a comprehensive European Union framework on endocrine disruptors.

### The document concludes as follows

Almost twenty years after the Community Strategy for endocrine disruptors of 1999, endocrine disruption remains a global challenge and a source of concern for many EU citizens. While significant progress has been achieved over the past two decades to better understand and manage endocrine disruptors, it is important to step up the EU’s efforts.

### The Commission further states

In order to be able to progress in effectively addressing endocrine disruptors, the Commission will follow an inclusive approach that is open, transparent and brings together all interested parties. The Commission stands ready to listen thoroughly, dialogue cooperatively and communicate proactively.

We appreciate the Commission’s intention of listening to all parties, including the scientific community. As a group of senior scientists with a long interest in this subject (Dietrich et al. 2013a, b, 2016; van Ravenzwaay et al. 2013; Autrup et al. 2015, 2016a, b; Gori and Dekant 2016) we respond to the Commission’s invitation to comment.

Accepting EFSA’s definition of “endocrine disruptors” (EFSA 2013) as chemicals capable of inducing endocrine-related effects in humans and animals,^1^ we highlight several basic concepts of toxicology that are essential for a comprehensive assessment of the regulatory framework of endocrine disruptors. These are:

1. Feed-back mechanisms of the endocrine system,
2. Biochemical principles of interaction at the level of receptors or enzymes,
3. Potencies of endogenous hormones, pharmaceutical drugs, phytoestrogens and S-EDCs,
4. Potential harmful effects of synthetic EDCs (S-EDCs),
5. Exposure to S-EDSs versus exposure to natural endocrine disruptors (N-EDCs),^2^
6. Conclusions and recommendation to evaluate potential risks of human exposure to S-EDCs.

## Feed-back mechanisms of the endocrine system

The function of the endocrine system is strictly regulated involving the hypothalamic/pituitary/gonad axis. The hypothalamus secretes stimulating and inhibiting factors that modulate the pituitary secretion of hormones. These then regulate diverse processes like the control of growth, metabolism, or reproductive cycles. The homeostasis of the endocrine system is regulated by feed-back mechanisms. The more common negative feed-back cycles negatively affect stimulation from a preceding tissue. The less common positive-feedback mechanisms positively affect or increase stimulation from the preceding tissue.

Estradiol and progesterone—both estrogen-based hormones—participate in both positive and negative feedback mechanisms within the female ovarian tissue. In short, any decrease in a hormone level at the specific target will result in an increased production and input of the specific hormone and vice versa.

The endocrine system can be modulated in two basic ways: (1) by agonists or antagonists of the respective estrogen and androgen receptors and (2) by interference with steroid biosynthesis and metabolism such as the conversion of testosterone to estrogen by aromatase followed by the conversion of testosterone to the more potent dihydrotestosterone by 5α-reductase.

For both, the interaction at a receptor and/or interference with a biosynthetic enzyme are biochemical processes that follow the laws of mass action. As a consequence, only exogenous ligands with high potency (high affinity to the receptor and in case of agonists, intrinsic efficacy of the ligand) and sufficiently high exposure at the target site can interfere with the function of endogenous hormones at receptors or enzymes.

The multiple growth promoting signals generated by an activated estrogen receptor (ER) includes stimulation of epidermal growth factors. Vice versa, epidermal growth factors can stimulate ER transcriptional activity. This cross-talk between epidermal growth factor receptors (EGFRs) and ERs specifically occurs in conjunction with EGFR overexpression in endocrine related cancer explaining resistance to hormone therapy (Collins et al. 2017). However, these cross-talk mechanisms are unlikely to occur at the exposure to S-EDCs observed for the general population.

## Biochemical principles of interaction at receptors or enzymes

Receptors are cellular components which bind molecules of diverse chemical structures. Known as ligands, these molecules activate or inhibit the receptor function and thereby elicit a physiological response. Ligands that activate a response are agonists; those that block the response are antagonists. Potency of the EDs depends on the strength of interaction of their ligand molecules with a specific receptor or enzyme.

Classes of receptors are various hormone- and neurotransmitter-receptors. The specific binding of a ligand at its receptor is a prerequisite for its action and triggers a cascade of events. The receptor ligand interaction follows the law of mass action and its kinetics are similar to the Michaelis Menten equilibrium except that the products of the Michaelis Menten type of interaction are metabolites whereas interactions of the agonist at the receptor usually do not result in a change of chemical structure of the agonist. Interaction of a ligand with a receptor is described by the equilibrium:

$${\text{ligand}}\, + \,{\text{receptor }} \rightleftarrows {\text{ligand}} - {\text{receptor complex}}$$

Replacement of a physiological ligand, such as a receptor bound estrogen, depends on the affinity of the receptor for that compound and its concentration at the receptor site. For example, partial replacement of the physiological ligand from the receptor by a compound of 1000-fold lower affinity requires a 1000-fold higher concentration than the endogenous compound. Although this oversimplifies competitive interaction of compounds at a receptor, it demonstrates the need for information on the relative binding affinities of the compounds in question and their concentration at the receptor. The same applies to the interference of a compound with an enzyme such as the specific inhibition of cytochrome P450 enzymes in the catabolism of retinoic acid by triazole fungicides (Menegola et al. 2006).

Based on these basic biochemical principles, (Borgert et al. 2013) concluded that a potency threshold exists for hormone-active compounds and that the manifestation of a detectable hormonal response at the tissue and the physiologic level in humans or animals depends on whether:

(1) a sufficient number of specific receptors are occupied by ligand molecules of sufficient specificity and potency to induce individual cells to respond to a given hormonal signal and

(2) a sufficient number of cells need to respond to a given hormonal stimulus to manifest a detectable physiologic effect at the tissue or organism level.

This has been exemplified by the case of diethylstilbestrol (DES), a synthetic non-steroidal selective estrogen receptor modulator (SERM), whose potency is equivalent to or greater than that of ethinyl estradiol (Borgert et al. 2012). In the 1950s and 1960s, DES was prescribed to large numbers of pregnant women at massive doses to prevent spontaneous abortions. The administered doses ranged from 5 mg/day up to 125 mg/day (approximately 2 mg/kg-bw/day).

DES-exposure in utero has increased the incidence of a rare tumor in young women and induced reproductive tract anomalies in males exposed in utero during critical phases of development based on the hormonal activity of DES. Thus, DES studies provide important data on dose–effect relationships in humans. Marked differences in DES dosing schedules used resulted in different effects in males prenatally exposed to DES. No indications for adverse consequences have been observed at comparatively low total maternal doses of approx. 1.4 g (sum of all doses during gestation) while adverse consequences have been observed at the high total DES dose of approx. 11.6 g. These human data demonstrate the existence of maternal dose levels below which adverse non-cancer effects do not occur. The extensive rodent DES reproductive toxicity data base is also consistent with this finding: non-cancer DES effects on fertility and genital tract abnormalities demonstrate dose levels below which adverse effects are not observed, i.e., dose response thresholds (Borgert et al. 2012). These fundamental principles are consistent with established knowledge about hormonal mechanisms with the obvious consequence that effects depend on potency and exposure (Autrup et al. 2016a).

Thus, if synthetic chemicals are to interfere with natural endocrine signals, their doses/concentrations and potencies need to be similar to or higher than those of natural hormones (Golden et al. 1998; Dietrich 2010; Marty et al. 2011). Otherwise, they cannot displace the numerous natural endogenous ligands present. This explains, for example, why S-EDCs with low relative potency have never been shown to exhibit estrogenic effects in humans (Borgert et al. 2018). Potency and concentrations define the minimum requirement for influencing endocrine activity. This implies that defining an endocrine hazard of EDCs (or a potential therapeutic effect) requires an evaluation of potency required for physiological activity as well as the physiologically achievable concentrations. These principles have successfully guided endocrine pharmacology (Cleve et al. 2012). Taking into account the mechanisms of hormone signalling and processing, safe levels of exposure can then be set for endocrine active substances based on basic biological and pharmacological principles (Borgert et al. 2012, 2013; Caldwell et al. 2012).

Although binding to the sex-hormone-binding-globulin may be relatively greater for the endogenous hormones than for N-EDCs and S-EDCs, it must be recognized that hormones are not the only endogenous ligands for hormone receptors. For example, dehydroepiandrosterone (DHEA) and its metabolites DHEA-sulfate, androstenedione, and androstenediol are endogenous, naturally occurring products of human metabolism that exhibit greater affinity and efficacy for the estrogen receptor than most chemicals claimed to be S-EDCs. These natural ligands are present in the blood at concentrations far greater than S-EDCs with concentrations ranging from picomolar to almost micromolar (Miller et al. 2013). Because of their affinity and high concentration in the body, these natural, endogenous ligands would occupy a significant fraction of any estrogen receptors not occupied by the endogenous hormones. Natural ligands also exist for other hormone receptors.

## Potencies of endogenous hormones, drugs, N-EDCs and S-EDCs

Endogenous hormones have to have a high affinity for their target receptors to effectively regulate physiological functions. Their affinities are much higher as compared to affinities of N-EDCs and S-ECCs. As outlined below, this is well known for more than 25 years.

In 1999, the Scientific Committee on Toxicology, Ecotoxicology and the Environment (EU-SCSTEE, 1999) published an opinion on the effects of endocrine disrupting chemicals on human and wildlife health. The opinion listed numerous reports on the concentration of EDCs in human food and tissues and on the relative potency of these chemicals in vitro, as compared to 17β-estradiol. In assessing the relative risk of EDCs, human exposures to these chemicals—assessed by their concentrations in blood or serum—were related to their estrogenic activity, determined in vitro as the concentration needed to attain 50% or 100% of maximum estrogenic activity.

Data on estrogen activities have been taken from different experimental approaches, such as competitive binding to recombinant human estrogen receptor of MCF-7 cells, proliferation of MCF-7 human breast cancer cells (E-SCREEN) or expression of a reporter gene in the yeast estrogen system (YES). The results of these assays showed that the relative in vitro potencies of *o,p*’-DDT, *p,p*’-DDT, PCBs, 4-nonylphenol, bisphenol A and dieldrin are several orders of magnitude lower than that of 17β-estradiol. The phytoestrogen genistein present in soy-based food at hgh concentrations had a higher potency (estrogen receptor binding affinity and intrinsic efficacy at the estrogen receptor) as compared to the investigated S-EDCs. Thus, it may exhibit estrogenic activity that exceeds the activity of circulating 17β-estradiol in persons who consume soy-rich diets. Genistein’s serum concentrations vary over a wide range in individuals consuming diets with varying soy content, leading to a wide range of possible estrogenic activity for this N-EDC.

In 2001, Leffers et al. (2001) compared the estrogenic potency of the synthetic estrogen zeranol, used as a growth promoter in meat production, and five related compounds, with the potency of 17β-estradiol, DES, genistein, and bisphenol A (BPA). Potency was assessed by analysing differences in expression levels of endogenous estrogen- regulated genes in human MCF7 cells. Zeranol, 17β-estradiol and DES had approximately equal potency. Genistein was four to six orders of magnitude less potent than 17β-estradiol but still an order of magnitude more potent than BPA. The very high potency of zeranol compared to the other potential endocrine disrupters suggests that zeranol intake from beef products may have a greater impact on consumers than the amounts of the known or suspected S-EDCs (e.g., BPA, DEHP, *o,p*’-DDT, PCPs, nonylphenol, dieldrin) present in food. The authors recommend reliable measurements of the concentration of zeranol in human serum after ingestion of meat products from treated animals, because zeranol is consumed in doses that may actually have hormonal activities.

A recent comparison of bisphenol A (BPA) and bisphenol F (BPF) that naturally occurs in sweet mustard demonstrated similar estrogenic potencies (Dietrich and Hengstler, 2016).

In addition to the studies of (Golden et al. 2005), (Witorsch 2002), (Witorsch and Thomas 2010) who demonstrated that natural or synthetic hormones such as ethinyl estradiol are 10,000 to 1,000,000 fold more potent than S-EDCs, (Nohynek et al. 2013) compared the estrogenic potencies of ethinyl estradiol (1,000,000), coumestrol (10,000), genistein (37), butylparaben (0.5) and benzylpareben (0.1) in the rodent uterotrophic assay (Table 1) (Nilsson 2000; Golden et al. 2005; Witorsch and Thomas 2010).

**Table 1:** Comparative potency of endogenous hormones, estrogenic drugs and some N- and S-EDCs

Substance	Use/origin	Effective dose (mg/kg/day)	Relative potency to 17β-estradiol
Diethylstilbestrol (DES)	Drug	0.0001	3,000,000
Ethinyl estradiol	Contraceptive	0.0003	1,000,000
Estrone	Human estrogen	0.0012	250,000
Coumestrol	Legumes	0.03	10,000
Genistein	Soybeans	8	37
Daidzein	Soybeans	12	25
4-MBC	UV filter	300	1.0
Butyl paraben	Preservative	600^a^	0.5
Benzyl paraben	Preservative	2500	0.12

^a^Subcutaneous 1 × 800 mg/kg, rats

As presented in the 2007 NTP-CERHR Expert Panel Report on BPA (Chapin et al. 2007), concentrations of BPA in the blood of German, US and Japanese pregnant women average between 0.43 and 4.4 μg BPA/l with individual concentrations between 0.2 and 18.9 μg/l. The relative estrogenic potency BPA is approximately 570 to 5800-fold lower that of 17β-estradiol. Even at the highest measured blood concentration of 18.9 μg BPA/l, BPA will produce an approx. 125 times lower estrogenic activity than the circulating levels of 17β-estradiol. The 2007 NTP-CEHR Report concluded that an interaction of BPA at the estrogen receptor with causal physiological consequences is unlikely. It should be mentioned that the blood values represent total BPA, but BPA in blood is mostly present in form of conjugates with a much lower estrogenic potency than the free BPA. Thus, estrogenic effects are expected to be even lower.

Bolt et al. (2001) compared the relative potencies of BPA and nonylphenol to those of daidzein and ethinyl estradiol. On the basis of comparative data from uterotrophic assays in rats, with three consecutive days of oral applications, and taking N-EDC daidzein as reference, relative uterotrophic activities in rats followed the sequence: daidzein = 1; BPA = 1; *p*-tert- octylphenol = 2; *o,p*'-DDT = 4; ethinyl estradiol = 40,000.

(Rietjens et al. 2017) assembled the results from studies on the competitive binding of 17β-estradiol and phytoestrogens to the ERα and ERβ receptors. The overall conclusion was that phytoestrogens were about 1000–10,000 times less potent estrogens than 17β-estradiol at both receptors.

These findings clearly indicate that S-EDCs and N-EDCs have a much lower potency than drugs designed to pharmacologically interfere with the endocrine system and that the potencies of S-EDCs (e.g., BPA) are similar or lower than those of N-EDCs (e.g., BPF). Remarkably, the intake of the highly potent ethinyl estradiol (EE) for contraception of young and middle-aged females is not questioned as a potential issue regarding EDCs although the potency of EE is about 100,000-fold higher than that of S-EDCs or N-EDCs. In summary, these observations do not support legislative attempts aiming to protect consumers from adverse effects focusing on S-EDCs while ignoring the significant human exposures to N-EDCs.

## Potential harmful effects of S-EDCs in humans

During the past decades, particular focus has been given to the potential harmful effects of EDCs to the reproductive system of humans based on epidemiological studies.

Sifakis et al. (2017) evaluated the available epidemiological studies on the effects of S-EDCs in humans and concluded that due to the complexity of the clinical protocols, the degree of occupational and environmental exposure, the variable endpoints measured, and sample sizes, causal relationships between the reproductive disorders and exposure to specific toxicants (S-EDCs) are not established.

Minguez-Alarcon and Gaskins (2017) summarized the epidemiological literature on the potential effects of female exposure to non-persistent S-EDCs including BPA, phthalates, parabens, and triclosan, on fecundity, measured by markers of reproductive hormones, markers of ovulation or ovarian reserve, in vitro fertilization outcomes, and time to pregnancy. They conclude that the heterogeneous results obtained could be due to methodological differences in the recruitment of participants (fertile vs. subfertile), study designs (prospective vs. retrospective), exposure assessment (including differences in the number and timing of urine samples and differences in the analytical methods used to assess the urinary concentrations), residual confounding factors due to diet or other lifestyle factors, and co-exposures to other chemicals.

Zamkowska et al. (2018) evaluated the vast current epidemiological literature on environmental exposure to S-EDCs and semen quality. Out of 970 references, only 45 articles met their quality criteria and were included. These studies provided data on sperm quality and biomonitoring-based exposure assessment for BPA, triclosan, parabens, synthetic pyrethroids, organophosphate pesticides and phthalates. The authors conclude that despite the numerous limitations of the results, the studies could suggest that exposure to the various compounds may be associated with affected semen quality parameters. However, due to the insufficiently solid evidence further epidemiological studies were needed to confirm these findings.

The same group (Karwacka et al. 2019) evaluated the available literature on S-EDCs exposure and their effect on the reproductive potential of women. The studies comprised prospective cohorts with exposure assessments based on concentrations in biological fluids including urine, serum, saliva. The S-EDCs included BPA, triclosan, parabens, phthalates, perfluorinated chemicals, polychlorinated biphenyls and organochlorine pesticides. The concentrations reported ranged between ≤ 1 ng/ml to a few μg/ml and the authors concluded that the evidence supporting an association between ECDs concentration and capacity of the ovary to provide egg cells capable for fertilization and in vitro fertilization outcomes in humans remains limited.

In a comprehensive review, Rietjens et al. (2017) evaluated the potential health effects of dietary phytoestrogens. The structural similarity to 17β-estradiol enables phytoestrogens to induce (anti) estrogenic effects by binding to the estrogen receptors (vide supra). Various beneficial health effects have been ascribed to phytoestrogen intake, e.g., a lowered risk of menopausal symptoms like hot flushes and osteoporosis, lowered risks of cardiovascular disease, obesity, metabolic syndrome and type 2 diabetes, brain function disorders, breast cancer, prostate cancer, bowel cancer and other cancers. However, the (anti) estrogenic properties of phytoestrogens also raised concerns that they might act as N-EDCs, thus having a potential to cause adverse health effects. The latter is somewhat of a misconception as the beneficial effects of phytoestrogens noted can clearly be ascribed to their endocrine activity, meaning that their beneficial effects should be considered as a consequence of their capabilities to affect the endocrine system. The literature overview presented illustrates that several potential health benefits of phytoestrogens have been reported but that, given the data on potential adverse health effects, the current evidence on these beneficial health effects is so obvious that they clearly outweigh the possible health risks. Furthermore, the data currently available are not sufficient to support a more refined (semi) quantitative risk-benefit analysis.

The serious drawback of all these studies is that while the mere presence of S-EDCs (in food or in humans based on biomonitoring) is considered to be a risk, the actual extent of EDC exposure is not discussed in context with the confounding exposure to N-EDCs. Due to the low potencies and exposures of S-EDCs as compared to high potencies of drugs with estrogenic activity and high exposures to N-EDCs, it has to be expected that studies which investigated the association between S-EDCs exposure and human health remain inconclusive. It also needs to be noted that exposures to S-EDCs has continuously declined over the past five decades while exposure to N-EDCs has increased (vide infra), primarily in conjunction with an increase in vegetarian lifestyles. Consequently, it is to be expected that future epidemiological studies on the adverse health effects of S-EDCs will have an ever-decreasing chance in associating exposure to S-EDCs to specific health effects when simultaneously ignoring the increasing exposures to N-EDCs. Thus, based on the low exposures and low potencies of S-EDCs the only biologically plausible and scientifically reasonable conclusion is that there is no association. Accordingly, Swaen et al. (2018), who evaluated the causes for the changing trends in possibly endocrine related diseases in the Western world, which are thought to originate from exposure to endocrine disruptors, concluded: Factors such as paternal age and maternal age at first pregnancy and parity explain a substantial proportion of the reported increases. Other factors such as BMI may play a similar role in the observed trend (Smith et al. Regul Toxicol Pharmacol in press).

## Exposure of synthetic EDCs versus natural EDCs

An array of information adds to the evidence that the daily intake of natural EDCs greatly exceeds that of S-EDCs e.g., (Safe 1995, 2000; Bolt et al. 2001; Dekant and Colnot 2013). The intake of phytoestrogens from food varies widely among different populations (British < 1 mg/day, in Asian countries up to 100 mg/day), depending on their dietary habits (Cassidy 1998).

Early on in the debate, Safe (2000) calculated the daily human intake of estrogen and anti-estrogenic equivalents, based on potencies of N-EDCs and S-EDCs relative to 17β-estradiol. It was calculated that a woman taking a birth control pill ingests about 16,675 μg of 17β-estradiol equivalents/day, postmenopausal estrogen therapy amounts to 3350 μg, ingestion of estrogen flavonoids in food represents 102 μg, whereas daily ingestion of environmental organochlorine-based S-EDCs considered relevant at this time was calculated to be 0.0000025 μg 17β-estradiol equivalents.

Patisaul and Jefferson (2010) evaluated the intake of flavones and other phytoestrogens in human diets after the US Food and Drug administration approved the health claim that daily consumption of soy is effective in reducing the risk of coronary artery disease. Since most phytoestrogens are phenolic compounds, with isoflavonoids and coumestans as major constituents, the authors specifically evaluated the daily intakes of genistein, daidzein and total isoflavones. Soy is abundant in traditional Asian diets that may result in isoflavonoid consumption up to daily doses of 50 mg/kg body weight. In the US, consumption of isoflavonoids ranges from 1 to 3 mg/kg when consuming “Western” diet, but a vegetarian lifestyle or use of soy-containing dietary supplements may result in intakes at or above levels seen in Asia. High daily doses of N-EDCs also occur in infants. For example, a dose of 6–9 mg total isoflavonoids/kg/day and genistein plasma levels up to 1000 ng/ml were seen in 4-month-old infants exclusively fed soy-based formula. In Asian women, blood genistein levels are in the range of 25 ng/ml and under 2 ng/ml for US women.

According to Bolt et al. (2001) who compiled the daily exposure data from the existing literature the daily exposures to N-EDCs (phytoestrogens) are: 4.5–8 mg/kg for infants on soy-based formula, 1–3 mg/kg for adults (western population), 50–100 mg for the East Asian population. By contrast, dietary exposures to individual S-EDCs are about 1000-fold lower.

Irvine et al. (1998) investigated the concentrations, daily intake and possible biological effects of phytoestrogens in infants, related to intake of increasingly popular soy-based food. Initially, the total amounts of genistein and daidzein in commercial soy-based infant formulas, infant cereals, dinners, and biscuits were measured. Phytoestrogens in dairy-based formulas and in breast milk from omnivorous or vegetarian mothers were also assessed. The phytoestrogen content of cereals varied, with a range of 3–287 μg genistein/g and 2–276 μg daidzein/g. When consumed according to the recommendations, soy formulas provide the infant with a daily dose rate of total isoflavones (genistein + daidzein) of approximately 3 mg/kg body weight between 0 and 4 months of age. Supplementing the diet of 4-month-old infants with a single daily serving of cereal can increase their isoflavone intake by over 25%. This isoflavone intake is much greater than in adults. Since infants can digest and absorb dietary phytoestrogens in active forms and neonates are generally more susceptible than adults to perturbations of the steroid equilibrium, Irvine et al. (1998) suggested that it is highly desirable to study the effects of soy isoflavones on steroid-dependent developmental processes in human babies.

In addition, the intake of N-EDCs may be higher for menopausal women who consume soy-based preparations as an alternative to steroid hormones. Isoflavone dose suggestions listed on marketed packages vary between 20 and 80 mg isoflavone per day. Moreover, prenyl flavonoids can be found in hops and end up in beer. High concentrations of coumestans are found in legumes and clover sprouts. Lignans are formed from lignan precursors by intestinal bacteria. Lignans are formed by intestinal bacteria from lignan precursors found in flaxseeds, whole grains, fruits and vegetables, sesame seeds and legumes all adding to the human body burden of N-EDCs.

These and an array of other studies show that human exposure to N-EDCs to be several orders of magnitude higher than to S-EDCs. In contrast, the daily intake of most S-EDCs is significantly lower, e.g., that of BPA is approximately 35 ng/kg/day, i.e., a factor 3000 lower than that of isoflavonoids. Despite these much higher exposures, a definite conclusion on putative beneficial or adverse effects of N-EDCs in humans remains elusive, further reinforcing the lack of evidence for adverse effects of S-EDCs, owing to their much lower exposures and potencies.

## Conclusion and recommendation to evaluate the risks of human exposure to S-EDCs

As outlined above, the potencies of S-EDCs are much lower than for N-EDCs, drugs or endogenous hormones. Therefore, at the low human exposures that have been demonstrated in all sensibly conducted studies, S-EDCs have virtually no chance to physiologically compete with natural hormones in binding to free receptors. This implies that the health risks of the known S-EDCs are nil or at least negligible. On these grounds and with the conservative assumption of similar endocrine mechanisms for S-EDCs, N-EDC and endogenous hormones, it is proposed to compare S-EDCs potencies with standard N-EDCs using appropriate in vitro test systems. Selection of the reference N-EDCs should be based on their potencies compared to the corresponding physiological hormones. When the potency of an S-EDC is similar or lower than for the N-EDC standard, further studies and regulatory consequences will not be warranted.

Such an in vitro evaluation would also overcome the concern expressed in the “Memorandum on endocrine disruptors” of the Scientific Committee on Consumer Safety (EU-SCCS 2014) as follows:

Due to the ban on animal testing for cosmetic ingredients effective since 2013, it will be extremely difficult in the future to differentiate between a potential ED and an ED, if the substance is registered solely for use in cosmetic products [Factsheet ECHA-14-FS-04- EN, https://echa.europa.eu/documents/10162/13628/reach_cosmetics_factsheet_en.pdf]. Yet, for substances registered under REACH and also for other (mixed) uses, crucial information from animal tests is necessary for the time being.

The replacement of animal test methods by alternative methods in relation to complex toxicological endpoints remains scientifically difficult, despite the additional efforts launched at various levels [SCCS/1294/10, Adler et al. 2011]. With regard to substances with endocrine activity (potential endocrine disruptors), the assessment of their impact to human health without animal data remains a challenge.

This Editorial represents a consensus of several editors and is being published simultaneously in *Chemico-Biological Interactions*, *Computational Toxicology*, *Environmental Toxicology and Pharmacology*, *Food and Chemical Toxicology*, *Toxicology In Vitro*, *Toxicology Letters*, *Archives of Toxicology* and further journals in the field of Toxicology.

## Footnotes

1 It is to be noted that “endocrine disruptors” is not a scientific term. “Chemicals interfering with the endocrine system” better defines their specific effects.

2 Naturally occurring substances that can interfere with the activity of circulating endogenous hormones, but excluding the latter.

## References

[CR500] Adler S, Basketter D, Creton S, Pelkonen O, van Benthem J (2011). Alternative (non-animal) methods for cosmetics testing: current status and futureprospects-2010. Arch Toxicol.

[CR1] Autrup H, Barile FA, Blaauboer BJ, Degen GH, Dekant W, Dietrich D, Domingo JL, Gori GB, Greim H, Hengstler JG, Kacew S, Marquardt H, Pelkonen O, Savolainen K, Vermeulen NP (2015). Principles of pharmacology and toxicology also govern effects of chemicals on the endocrine system. Toxicol Sci.

[CR2] Autrup H, Barile FA, Blaauboer BJ, Degen GH, Dekant W, Dietrich D, Domingo JL, Gori GB, Greim H, Hengstler JG, Kacew S, Marquardt H, Pelkonen O, Savolainen K, Vermeulen NP (2016). Response to “the path forward on endocrine disruptors requires focus” by Zoeller, et al.. Toxicol Sci.

[CR3] Autrup HN, Berry SC, Cohen SM, Creppy EE, de Camargo JL, Dekant W, Dietrich D, Galli CL, Goodman JI, Gori GB, Greim HA, Klaunig JE, Lotti M, Marquardt HW, Wallace KB, Yamazaki H (2016). Whither the impending European regulation of presumed endocrine disruptors?. Regul Toxicol Pharmacol.

[CR4] Bolt HM, Janning P, Michna H, Degen GH (2001). Comparative assessment of endocrine modulators with oestrogenic activity: I. Definition of a hygiene-based margin of safety (HBMOS) for xeno-oestrogens against the background of European developments. Arch Toxicol.

[CR5] Borgert CJ, Baker SP, Matthews JC (2013). Potency matters: thresholds govern endocrine activity. Regul Toxicol Pharmacol.

[CR6] Borgert CJ, Matthews JC, Baker SP (2018). Human-relevant potency threshold (HRPT) for ERalpha agonism. Arch Toxicol.

[CR7] Borgert CJ, Sargent EV, Casella G, Dietrich DR, McCarty LS, Golden RJ (2012). The human relevant potency threshold: reducing uncertainty by human calibration of cumulative risk assessments. Regul Toxicol Pharmacol.

[CR8] Caldwell DJ, Mastrocco F, Anderson PD, Lange R, Sumpter JP (2012). Predicted-no-effect concentrations for the steroid estrogens estrone, 17beta-estradiol, estriol, and 17alpha-ethinylestradiol. Environ Toxicol Chem.

[CR9] Cassidy A (1998) Risks and benefits of phytoestrogen-rich diets. In Eisenbrand G Daniel H Dayan AD Elias PS Grunow W Kemper FH Löser E Metzler M J Schlatter (Eds) Symposium: hormonally active agents in food, organized by the DFG Senate Commission of Food Safety. Kaiserslautern, October 6–9th, pp 91–117

[CR33] Chapin RE, Adams J, Boekelheide K, Gray LE, Hayward SW, Lees PS, McIntyre BS, Portier KM, Schnorr TM, Selevan SG, Vandenbergh JG, Woskie SR (2007). NTP-CERHR expert panel report on the reproductive and developmental toxicity of Bisphenol A.

[CR10] Cleve A, Fritzemeier KH, Haendler B, Heinrich N, Moler C, Schwede W, Wintermantel T (2012). Pharmacology and clinical use of sex steroid hormone receptor modulators. Handb Exp Pharmacol.

[CR11] Collins D, Jacob W, Cejalvo JM, Ceppi M, James I, Hasmann M, Crown J, Cervantes A, Weisser M, Bossenmaier B (2017). Direct estrogen receptor (ER)/HER family crosstalk mediating sensitivity to lumretuzumab and pertuzumab in ER+ breast cancer. PLoS ONE.

[CR12] Dekant W, Colnot T (2013). Endocrine effects of chemicals: aspects of hazard identification and human health risk assessment. Toxicol Lett.

[CR13] Dietrich D, Hengstler J (2016). From bisphenol A to bisphenol F and a ban of mustard due to chronic low-dose exposures?. Arch Toxicol.

[CR14] Dietrich D, von Aulock S, Marquardt HW, Blaauboer BJ, Dekant W, Kehrer J, Hengstler JG, Collier AC, Gori GB, Pelkonen O, Lang F, Nijkamp FP, Stemmer K, Li A, Savolainen K, Hayes AW, Gooderham N, Harvey A (2013). Open letter to the European Commission: scientifically unfounded precaution drives European Commission’s recommendations on EDC regulation, while defying common sense, well-established science, and risk assessment principles. Arch Toxicol.

[CR15] Dietrich DR (2010). Courage for simplification and imperfection in the 21st century assessment of “Endocrine disruption”. Altex.

[CR16] Dietrich DR, Dekant W, Greim H, Heslop-Harrison P, Berry SC, Boobis A, Hengstler JG, Sharpe R (2016). Allowing pseudoscience into EU risk assessment processes is eroding public trust in science experts and in science as a whole: the bigger picture. Chem Biol Interact.

[CR17] Dietrich DR, von Aulock S, Marquardt H, Blaauboer B, Dekant W, Hengstler JG, Collier A, Gori GB, Pelkonen O, Lang F, Nijkamp FP, Stemmer K, Li A, Savolainen K, Hayes AW, Gooderham N, Harvey A (2013). Editorial. Regul Toxicol Pharmacol.

[CR18] EFSA (2013). Scientific Opinion on the hazard assessment of endocrine disruptors: Scientific criteria for identification of endocrine disruptors and appropriateness of existing test methods for assessing effects mediated by these substances on human health and the environment. EFSA J.

[CR19] EU-SCCS (2014) Memorandum on endocrine disruptors of the scientific committee on consumer safety (SCCS) adopted at its 8th plenary meeting on 16 December 2014 (SCCS/1544/14). 10.2772/52077

[CR20] EU-SCSTEEn (1999) CSTEE opinion on human and wildlife health effects of endocrine disrupting chemicals, with emphasis on wildlife and on a ecotoxicology test methods expressed at the 8th CSTEE plenary meeting, Brussels, 4 March 1999. Report of the working group on endocrine disrupters of the scientific committee on toxicity, ecotoxicity and the environment (CSTEE) of DG XXIV, consumer policy and consumer health protection

[CR21] Golden R, Gandy J, Vollmer G (2005). A review of the endocrine activity of parabens and implications for potential risks to human health. Crit Rev Toxicol.

[CR22] Golden RJ, Noller KL, Titus-Ernstoff L, Kaufman RH, Mittendorf R, Stillman R, Reese EA (1998). Environmental endocrine modulators and human health: an assessment of the biological evidence. Crit Rev Toxicol.

[CR23] Gori GB, Dekant W (2016). Deepening uncertainty on how the EU may regulate supposable endocrine disruptors. Regul Toxicol Pharmacol.

[CR24] Irvine CH, Fitzpatrick MG, Alexander SL (1998). Phytoestrogens in soy-based infant foods: concentrations, daily intake, and possible biological effects. Proc Soc Exp Biol Med.

[CR25] Karwacka A, Zamkowska D, Radwan M, Jurewicz J (2019). Exposure to modern, widespread environmental endocrine disrupting chemicals and their effect on the reproductive potential of women: an overview of current epidemiological evidence. Hum Fertil (Camb).

[CR26] Leffers H, Naesby M, Vendelbo B, Skakkebaek NE, Jorgensen M (2001). Oestrogenic potencies of Zeranol, oestradiol, diethylstilboestrol, Bisphenol-A and genistein: implications for exposure assessment of potential endocrine disrupters. Hum Reprod.

[CR27] Marty MS, Carney EW, Rowlands JC (2011). Endocrine disruption: historical perspectives and its impact on the future of toxicology testing. Toxicol Sci.

[CR28] Menegola E, Broccia ML, Di Renzo F, Giavini E (2006). Postulated pathogenic pathway in triazole fungicide induced dysmorphogenic effects. Reprod Toxicol.

[CR29] Miller KK, Al-Rayyan N, Ivanova MM, Mattingly KA, Ripp SL, Klinge CM, Prough RA (2013). DHEA metabolites activate estrogen receptors alpha and beta. Steroids.

[CR30] Minguez-Alarcon L, Gaskins AJ (2017). Female exposure to endocrine disrupting chemicals and fecundity: a review. Curr Opin Obstet Gynecol.

[CR31] Nilsson R (2000). Endocrine modulators in the food chain and environment. Toxicol Pathol.

[CR32] Nohynek GJ, Borgert CJ, Dietrich D, Rozman KK (2013). Endocrine disruption: fact or urban legend?. Toxicol Lett.

[CR34] Patisaul HB, Jefferson W (2010). The pros and cons of phytoestrogens. Front Neuroendocrinol.

[CR35] Rietjens I, Louisse J, Beekmann K (2017). The potential health effects of dietary phytoestrogens. Br J Pharmacol.

[CR36] Safe SH (1995). Environmental and dietary estrogens and human health: is there a problem?. Environ Health Perspect.

[CR37] Safe SH (2000). Endocrine disruptors and human health—is there a problem? An update. Environ Health Perspect.

[CR38] Sifakis S, Androutsopoulos VP, Tsatsakis AM, Spandidos DA (2017). Human exposure to endocrine disrupting chemicals: effects on the male and female reproductive systems. Environ Toxicol Pharmacol.

[CR39] Smith CJ, Perfetti TA, Hayes AW, Berry SC. Clinical epidemiology studies on potential effects of endocrine disrupting chemicals should exclude obesity subjects. Regul Toxicol Pharmacol (in press) 10.1016/j.yrtph.2020.104711 32598900

[CR40] Swaen GMH, Boffetta P, Zeegers M (2018). Impact of changes in human reproduction on the incidence of endocrine-related diseases. Crit Rev Toxicol.

[CR41] van Ravenzwaay B, Dekant W, Vrijhof H (2013). Risk assessment of endocrine disrupting chemicals–introduction to this special issue. Toxicol Lett.

[CR42] Witorsch RJ (2002). Endocrine disruptors: can biological effects and environmental risks be predicted?. Regul Toxicol Pharmacol.

[CR43] Witorsch RJ, Thomas JA (2010). Personal care products and endocrine disruption: a critical review of the literature. Crit Rev Toxicol.

[CR44] Zamkowska D, Karwacka A, Jurewicz J, Radwan M (2018). Environmental exposure to non-persistent endocrine disrupting chemicals and semen quality: an overview of the current epidemiological evidence. Int J Occup Med Environ Health.

